# Interdisciplinary oral and primary health care for patients with disabilities

**DOI:** 10.3389/fmed.2025.1619845

**Published:** 2025-07-17

**Authors:** Sodabeh Etminan, Elsa Hammerdahl, Linda Lesondak, Nicole Li, Manav Patel, Matthew Mischler, Mary Keehn, Kristi Kirschner

**Affiliations:** ^1^University of Illinois Chicago, UI Health, Mile Square Health Center, Chicago, IL, United States; ^2^University of Illinois Chicago, Chicago, IL, United States

**Keywords:** oral health, primary care, intellectual and developmental disability (IDD), interdisciplinary care, disability, access to care, federally qualified health center (FQHC)

## Abstract

**Introduction:**

Individuals with intellectual and developmental disabilities (IDD) often face access to care barriers due to limited provider training, inadequate appointment availability, transportation barriers, financial limitations, and insufficient interdisciplinary collaboration. As a result, this group is particularly vulnerable to oral health issues, experiencing higher rates of periodontal disease and dental caries compared to those without disabilities. Survey data from both dentists and caregivers highlights these obstacles, revealing that a significant proportion of providers do not treat individuals with cognitive disabilities, and patients with IDD often seek care on an emergency basis. This study explores whether a specialized model of dental care—featuring longer appointment times, desensitization techniques, accessible dental furniture, and an interdisciplinary, collaborative team of providers trained to work with individuals with disabilities—can improve dental and overall health outcomes.

**Methods:**

A total of 50 participants aged 18 years or older with an intellectual or developmental disability (IDD) diagnosis were recruited from a Federally Qualified Health Center (FQHC). Participants were identified by a physician specializing in IDD. Participants who consented were provided with an iPad to complete a 20-min electronic REDCap survey assessing their experiences at Mile Square Health Center, prior dental visits, and barriers to care.

**Results:**

Longer appointment times, accessible dental furniture, and providers with training in treating patients with IDD appeared to significantly improve the patient experience.

**Discussion:**

The findings suggest that longer appointment times, a non-coercive approach, and improved referral and transportation pathways can positively enhance both treatment outcomes and patient satisfaction. This study also emphasizes the importance of interdisciplinary collaboration in developing referral pathways and integration of dental care within the broader healthcare services for individuals with IDD as a model care delivery system.

## Introduction

Individuals with intellectual and developmental disabilities (IDD) often face significant obstacles in accessing comprehensive healthcare, including both oral and primary care (1, 2). This group is particularly vulnerable to oral health issues, experiencing higher rates of periodontal disease and dental caries compared to those without disabilities (2, 3). These challenges stem from various factors, such as limited provider training, inadequate appointment availability, transportation barriers, financial limitations, and insufficient interdisciplinary collaboration (4, 5). Communication barriers, in particular, complicate the situation, as many patients with IDD have difficulty expressing their needs or understanding healthcare instructions (4, 6). In dental settings, where cooperation is key to successful treatment, these barriers are even more pronounced, with patients often exhibiting avoidance behaviors due to fear or discomfort (7, 8). Survey data from both dentists and caregivers reinforce these challenges, revealing that a significant proportion of providers do not treat individuals with cognitive disabilities, and patients often delay care until emergencies arise (9). Addressing these unique needs requires a tailored approach to healthcare delivery that ensures equitable access and quality outcomes for this population. This study explores whether a specialized model of dental care—featuring longer appointment times, desensitization techniques, accessible dental furniture, and providers trained to work with individuals with disabilities - can improve dental outcomes and patient satisfaction.

Given these challenges, one key area of focus in our research is to improve appointment structures to better accommodate the time and care required for patients with IDD. Collaborations with specialty doctors and healthcare stakeholders are critical in creating refined referral pathways and expanding appointment availability (10, 11). Our approach enables a more seamless integration of primary and specialty care for patients with IDD (10, 11). Additionally, the project incorporates transportation assistance programs to address a common barrier for patients (4, 12). These adjustments aim to provide care teams with the necessary time to gather thorough patient histories, communicate effectively, and develop personalized treatment plans, ultimately ensuring better outcomes for individuals with IDD.

Another key component of our study was to address the educational gaps among healthcare providers. By integrating targeted training opportunities, such as supervised clinical experiences and interdisciplinary lectures, medical and dental students gain valuable skills and confidence in treating patients with disabilities (11, 12). Ongoing training is essential for healthcare providers to effectively address the specific challenges of treating this population (13, 14). Workforce shortages, recruitment challenges, and provider burnout have been identified as critical issues affecting healthcare access, underscoring the need for targeted training programs (14–16). Ultimately, these efforts aim to build a more inclusive, well-prepared healthcare workforce capable of delivering high-quality care to individuals with IDD, ensuring they receive the specialized treatment they deserve.

Building on the efforts to create a more inclusive healthcare workforce, the study prioritized the perspectives and feedback of patients and caregivers. Through structured surveys and follow-up consultations, we gathered insights into the healthcare experiences of individuals with IDD and their families (11, 17). This feedback informed the development of practical, patient-centered solutions that can be implemented across healthcare settings (18–20). By combining interdisciplinary collaboration, continued care, provider education, and patient input, this research aims to establish a sustainable model for improving healthcare accessibility and outcomes for individuals with IDD (1, 19, 20).

The overall aim of the project was to improve the oral healthcare experience for individuals with IDD in a federally qualified health center (FQHC) that includes an integrated dental setting. Medical, dental, and behavioral health services are Mile Square Health Center are co-located within the same facility and share a common waiting area. This integrated setup supports coordinated care and allows for more efficient appointment scheduling across service lines. Internal communication among clinic staff helps ensure that patients receive timely and streamlined access to multiple services during a single visit when possible. By focusing on patient-reported experiences, outcomes, and feedback on appointment structure, the study assessed the effectiveness of a holistic, patient-centered approach to dental care. We specifically explored whether a tailored model of care - featuring longer appointment times, desensitization techniques, accessible dental furniture, and providers trained to work with individuals with disabilities - could improve dental outcomes and patient satisfaction. Additionally, this study identified key barriers and facilitators to achieving good oral health outcomes and aims to refine the care model to better meet the unique needs of this underserved population.

## Materials and methods

### Participants

A total of 50 participants aged 18 years or older with an intellectual or developmental disability (IDD) diagnosis were recruited from the Mile Square Health Center Auburn Gresham clinic. A majority of participants were identified by a physician and referred to the dental providers at the Auburn Gresham clinic for dental care. However, a smaller subset of participants sought dental services independently (without physician referral). A sample size of 50 in research is often chosen as a balance between practicality and statistical power. It’s large enough to provide some meaningful insights and potentially detect meaningful effects, but not so large as to make the study excessively costly or time-consuming. This size can be suitable for pilot studies, exploratory research, or when working with specific populations where larger samples are difficult to obtain such as this one (21).

### Procedures

Participants were recruited between October 2023 and November 2024 in person at the Mile Square Health Center Auburn Gresham Dental Clinic or via phone. Eligibility criteria included being 18 years or older and having a diagnosed IDD condition. Exclusion criteria included the inability to provide assent without an LAR present at the visit and the inability to travel to the clinic for care.

Participants were identified by their Primary Care Provider (PCP) or dental provider specializing in IDD and were approached immediately after their 75-min dental visit to establish care, this was 15-min longer than a standard visit to accommodate this population. Study personnel conducted the informed consent process in a private room. For participants requiring a Legally Authorized Representative (LAR), informed consent was obtained from the LAR, and assent was obtained from the participant. Participants who consented were provided an iPad to complete a 20-min electronic REDCap survey assessing their experiences at Mile Square Health Center, prior dental visits, and barriers to care. The survey questions were adapted from the past survey question literature (22, 23). LARs or caregivers assisted as needed, including selecting responses on the iPad when the participant was unable to do so independently.

For participants who had completed their dental visit before the study began, recruitment, consent, and survey completion occurred via phone. All participants received a $30 gift card to a local store.

### Retrospective chart review

A retrospective chart review was conducted both before and after survey completion. Before recruitment, medical records were reviewed to obtain personal identifiers (e.g., name, date of birth, phone number, and email) necessary for participant identification and outreach. No protected health information (PHI) was collected in the survey responses. Following survey completion, additional clinical data were extracted from EPIC and Dentrix, including dates of service, results of physical examinations, medical history, medication history, and dental X-rays.

### Data analytic plan

This observational study aimed to identify barriers to oral healthcare among IDD patients. Survey responses and clinical records were analyzed using descriptive statistics to summarize participant demographics and trends in healthcare experiences. Qualitative responses were examined using thematic analysis to categorize recurring themes in patient satisfaction, barriers to care, and accessibility improvements. Findings are presented in summary tables and figures.

## Results

### Population characteristics

The study sample included 50 individuals with intellectual and developmental disabilities (IDD), aged between 18 and 79 years. The majority of participants identified as male (68%), Black or African American (64%), and non-Hispanic or Latino (64%) (see Table 1 for full demographic details). The most prevalent forms of disability were communicative (66%), cognitive (60%), manual dexterity (60%), and mobility (48%), with 74% of participants indicating they were unable to make their own decisions.

**Table 1:** Demographic characteristics.

Characteristic	*N* = 50	
	*n*	%
Age		
18–29	28	56
30–39	16	32
40–49	1	2
50–59	1	2
60–79	4	8
Gender		
Male	34	68
Female	16	32
Race		
White or Caucasian	14	28
Black or African-American	32	64
Asian American/Asian	1	2
Native Hawaiian/Other Pacific Islander	1	2
Other	2	4
Ethnicity		
Hispanic or Latino	13	26
Not Hispanic or Latino	32	64
Unspecified	5	10

Of the 50 patients, 49 had at least one documented diagnosis listed in their medical problem list. While many diagnoses reflected core IDD conditions—such as autism, cerebral palsy, and developmental delay—a range of co-occurring conditions were also identified. These included *neurological conditions* such as epilepsy (*n* = 16), seizures (*n* = 7), Charcot–Marie–Tooth disease (*n* = 1), multiple sclerosis (*n* = 1), and paralysis (*n* = 1); *psychiatric conditions* such as schizophrenia or schizoaffective disorder (*n* = 2) and depression (*n* = 1); and *cardiovascular conditions* (*n* = 3). Additional co-occurring diagnoses included scoliosis (*n* = 3), genetic disorders (*n* = 2), asthma (*n* = 1), kidney disease (*n* = 1), and tumors (*n* = 1). Some patients had both epilepsy and seizures documented, while others had one or the other; thus, these categories were retained separately to avoid assumptions about overlap. Several participants had more than one co-occurring condition.

As a result of these complex medical and developmental needs, many patients reported requiring assistance in various aspects of dental care. Specifically, 92% of patients expressed the need for help attending dental appointments. The majority (70%) reported relying on another person for transportation, while 24% use a scheduled transportation service, and 6% relied on public transportation. Regarding daily oral hygiene, 74% of participants required assistance, with family and friends serving as the primary source of help for 91.9% of those individuals. At dental visits, nearly two-thirds of patients (62%) reported needing additional assistance or accommodations. Examples of these accommodations included sedation (Nitrous), accessibility resources (e.g., wheelchair access, larger examination rooms), and mobility-related assistance (e.g., sitting down, staying still). See Table 2 for a summary of assistance needs reported by patients during dental visits and for daily oral hygiene.

**Table 2:** Participant responses to survey items assessing their need for assistance to or during dental appointments and with daily oral hygiene.

Survey item	*N* = 50	
	*n*	*%*
Dental visit accompaniment		
Attends alone	4	8
Someone accompanies	46	92
Dental visit accommodations		
Yes	31	62
No	19	38
Specific types of visit accommodations^a^		
Sedation (nitrous)	3	11.11
Accessibility (wheelchair, large examination room, etc.)	4	14.81
Transportation/Mobility (sitting down, staying still/calm, etc.)	9	33.33
Communication	7	25.93
Guardian	2	7.41
Other	2	7.41
Mode of transportation		
With a scheduled transportation service	12	24
Driven by someone	35	70
Public transportation	3	6
Assistance with daily oral hygiene		
Yes	37	74
No	13	26
Who provides daily oral hygiene assistance^b^		
Someone who comes into the home or works in the living facility	3	8.1
Family or friends	34	91.9

a Participants responded to this question only if they indicated “yes” to needing additional assistance or accommodations during the visit. Of the 31 who responded “yes,” only 27 provided a written explanation.

b Participants responded to this question only if they indicated “yes” to needing assistance taking care of their teeth/mouth. Hence, the total number of respondents (N) was 37.

### Prior care

Prior to their care at Mile Square Health Center, participants reported significant barriers to accessing oral health care. Over half (58%) had not seen a dentist in over a year, with the most common reasons being accessibility issues. Seventeen patients (34%) identified being unable to find an accessible dentist office as a reason for not seeing a dentist in over a year. Similarly, 40% felt they were unable to find a dentist who knew how to treat them effectively due to their medical condition or disability. Eighty percent of participants reported challenges in finding care, with 50% unable to find a dentist where they could schedule an appointment and 30% having to make several attempts at scheduling before getting an appointment. Additionally, of the 49 who responded, approximately 65% reported they did not have a dentist they saw consistently. The most common frequencies of dental visits were every 6 months (31.3%) and other (29.2%). Satisfaction with prior care was overall low, with less than half of participants (46%) expressing satisfaction with their prior dental care and a little over a third (34%) indicating they could not answer. See Table 3 for a summary of participants’ responses to questions regarding prior care.

**Table 3:** Participant responses to survey items assessing participant barriers to care prior to Mile Square Health Center.

Survey item	*N* = 50	
	*n*	*%*
Prior dental visit		
Less than 6 months	7	14
Between 6 months and a year	8	16
More than a year ago	29	58
I do not remember	6	12
Dentist visit frequency^a^		
Only if a tooth hurts	3	6.3
Every 3 months	5	10.4
Every 6 months	15	31.3
Once a year	11	22.9
Other	14	29.2
Ease of finding a dentist		
Easy to find a dentist	10	20
Had to try making an appointment with several dentists before getting one	15	30
Never able to find a dentist where one could make an appointment	25	50
Have regular dentist^b^		
Yes	17	34.7
No	32	65.3
Reasons for not visiting the dentist (if over a year)^c^		
Unable to find an accessible office	17	34
Unable to find a dentist who knows how to treat disability	20	40
Dental care satisfaction		
Very satisfied	18	36
Satisfied	5	10
Dissatisfied	6	12
Very dissatisfied	4	8
Cannot answer	17	34

a Two participants did not respond to this item (*N* = 48).

b One participant did not respond to this item (*N* = 49).

c These questions were asked as a series of checkboxes. Only the two answers with the highest percentage of yes’s were reported here.

Alongside challenges in accessing dental care, many participants struggled with daily oral hygiene maintenance prior to coming to Mile Square. Only 50% of patients reported brushing their teeth twice a day, and few reported using tools for flossing (dental floss: 20%, dental floss sticks: 8%; waterpik: 8%).

### Care at Mile Square Health Center

After care, a majority of participants reported a positive experience receiving oral health care at Mile Square Health Center. When asked about aspects of the experience that were not helpful, 80% of participants who responded indicated there were none. Of the forty-nine who responded, approximately 88% indicated they were satisfied with their care. Patients highlighted several positive aspects of their care, including the staff’s communication, interpersonal manner (e.g., kindness, patience, empathy, etc.), and clinical skills (e.g., professionalism, knowledge, etc.), and the patient center-care (e.g., comfort and accommodations).

Longer appointment times, accessible dental furniture, and providers with training in treating patients with IDD appeared to significantly improve the patient experience. Of those who responded, 100% agreed or strongly agreed that the dentist at Mile Square understood how to care for patients with disabilities, and 100% agreed or strongly agreed that the dentist’s explanations during the appointment helped them understand the procedures. All respondents also indicated that the appointment duration was sufficient to receive care without feeling rushed. Additionally, 91.3% of respondents agreed or strongly agreed that the dental chair worked well for them. These results are summarized in Table 4. Agree and strongly agree responses were combined for reporting purposes to capture overall endorsement.

**Table 4:** Participant responses to five survey items assessing their experience receiving dental care at Mile Square Health Center.

Survey item	*N* = 50	
	*n*	*%*
Dentists’ knowledge of disability^a^		
Strongly agree	40	100
Agree	9	81.6
Disagree	0	0
Strongly disagree	0	0
Appointment length satisfaction^b^		
Strongly agree	35	74.5
Agree	12	25.5
Disagree	0	0
Strongly disagree	0	0
Dentist explanation satisfaction		
Strongly agree	40	80
Agree	10	20
Disagree	0	0
Strongly disagree	0	0
Dental chair functionality satisfaction^c^		
Strongly agree	33	71.7
Agree	9	19.6
Disagree	4	8.7
Strongly disagree	0	0
Overall satisfaction^a^		
Very satisfied	41	83.7
Satisfied	4	8.2
Dissatisfied	2	4.1
Very dissatisfied	0	0
Do not know	2	4.1

a One participant did not respond to this item (*N* = 49).

b Three participants did not respond to this item (*N* = 47).

c Four participants did not respond to this item (*N* = 46).

## Discussion

This study highlighted critical barriers to oral health care access among individuals with intellectual and developmental disabilities (IDD), as well as the impact of receiving care at Mile Square Health Center. It explored unique strategies to improve dental care experiences for individuals with IDD, a group that often faced significant barriers to oral healthcare access, including limited availability of trained providers, communication difficulties, anxiety, fear, transportation challenges, and difficulties in establishing positive relationships with healthcare providers (5, 13).

Placing a large emphasis on non-coercive care positively benefited the patient-provider relationship, creating a unique sense of trust in patients (24, 25). Many of these patients were either wheelchair-bound or had motor disabilities, and providing aid in transportation positively impacted patient care (26–28). Additionally, a large focus was placed on bridging education gaps among healthcare professionals to improve their ability to deliver patient-centered care (13, 29, 30). Through these interventions, patients were provided with the proper resources, time, and communication to ensure a positive dental care experience.

### Unique contributions of this study

Unlike many studies that primarily focused on provider training initiatives, this study placed an emphasis on direct modifications to patient experiences. Another key distinction was the integration of real-time patient and caregiver feedback, via a survey, allowing for a patient-centered perspective that created practical improvements in patient care delivery (31–33).

A particularly unique aspect to this study was the inclusion of a town hall meeting, which took place near the end of the data collection period (after a majority of surveys had already been collection). The purpose of this event was to create a virtual space for providers to engage with patients and their families outside of a traditional clinical setting. Three patient families attended and shared their experience with the implemented intervention- specifically, their experience receiving dental care at Mile Square Health Center. They reported numerous positive outcomes, including:

- Better oral health due to increased cooperation and patient understanding of their care.
- Enhanced oral health education, which helped patients maintain higher oral hygiene standards at home.
- Overall satisfaction and gratitude toward the study, with families expressing appreciation for the patient-centered approach provided in this study, which contributed to the overall positivity of care.
- Improved communication between patients, caregivers, and the provider.

By actively engaging patients and their families in the care process, participants were more enthusiastic about sharing their experiences. Although providers did not receive additional supervised or structured training outside of the town hall, the event offered crucial insights into patient and family perspectives. Dr. Sodabeh Etminan completed the required training and received the “Dental Care for Persons with Disabilities” certificate from the University of Pennsylvania School of Medicine and Dr. Sai Krishna Kumar is partway through the certificate program. These insights reinforced the importance of adapting care approaches for patients with intellectual and developmental disabilities (IDD). Furthermore, such modifications could be extended to other healthcare settings, potentially improving access to care for individuals with IDD across a broader range of services.

### Practical implications

The findings suggested that longer appointment times, a non-coercive approach, and improved referral and transportation pathways could positively enhance both treatment outcomes and patient satisfaction (34, 35). Implementing these strategies on a larger scale could help improve trust between patients and providers, reduce dental avoidance behaviors, aggressive behavior, and foster long-term oral health improvements. Additionally, this study emphasized the importance of interdisciplinary collaboration in developing referral pathways and integration of dental care within the broader healthcare services for individuals with IDD.

Another critical implication was the role of educating providers in addressing healthcare disparities. By providing targeted training opportunities for existing providers or healthcare students, such as supervised clinical experiences and interdisciplinary workshops, current and future healthcare professionals could develop the necessary skills and confidence to treat individuals with IDD (36, 37). Furthermore, the study highlighted the need for a continued education effort to ensure that providers were ready to deliver high-quality, compassionate care at all times.

### Limitations

Despite these positive and unique findings, the study has limitations. First, with a sample size of only 50 patients, these results may not fully capture the diversity of experiences and barriers faced by individuals with IDD (38). Additionally, this study was conducted in a Federally Qualified Health Center (FQHC), which may have introduced bias in participant feedback (39, 40). In addition, since the surveys were administered in the same setting where care was provided, participants may have felt compelled to provide more favorable responses. However, we took steps to mitigate this risk: surveys were conducted in a consult room separate from the clinical space, and participants interacted with research staff unaffiliated with their care.

Furthermore, because most participants were recruited through physician referrals, there is a potential selection bias favoring individuals already engaged in healthcare (41). It is important to note, that while these participants had access to medical care, the majority were not previously connected to dental services. This highlights the key strength of our intervention to bridge the gap between medical and dental care for individuals with IDD. Finally, there are many IDD populations, particularly in underserved or rural areas, who lack access to any form of healthcare. Future research should include larger and more diverse samples from varied geographic locations and should examine the long-term impact of such interventions on oral health outcomes (2, 37). These interventions could also be adapted and implemented across different healthcare settings to gain a broader understanding of their effects on access and care delivery for individuals with IDD.

## Data Availability

The raw data supporting the conclusions of this article will be made available by the authors, without undue reservation.
